# Predicting anti-cancer drug sensitivity through WRE-XGBoost algorithm with weighted feature selection

**DOI:** 10.1016/j.gendis.2024.101275

**Published:** 2024-03-22

**Authors:** Yiyao Jiang, Ming Chen, Zhongmin Xiong, Yufang Qin

**Affiliations:** a College of Information Technology, Shanghai Ocean University, Shanghai, 201306, China; b Key Laboratory of Fisheries Information Ministry of Agriculture, Shanghai, 201306, China

Accurate prediction of drug susceptibility is one of the most important steps in personalized medicine. Applications of machine learning to pharmacogenomic data for sensitivity prediction can help study the mechanism of drug response and find more effective anti-tumor drugs. At present, most machine learning algorithms for predicting the drug sensitivity of cancer cell lines involve gene-level characteristics. However, the auxiliary information of drugs has been proved to improve the prediction accuracy by providing a priori information for drug response. Here, we developed the WRE-XGBoost model, using gene expression of cell lines and drug properties as input, which consists of a weighted algorithm of the random forest regression and elastic net regression (WRE) to select the important features and an improved XGBoost algorithm associating with particle swarm optimization to predict cell viability (Fig. S1). The experimental results of our model were superior to frequently used machine learning methods through cross-validation.

First, we compared our feature selection method WRE with other two methods, random forest (RF) and SelectKBest (KBest). To evaluate the performance of WRE, RF, and KBest, we used Pearson's correlation coefficients and coefficient of determination (R^2^) as performance measures for the prediction of cell viability in the COSMIC-CTRP dataset (Fig. 1A, B). Synthetically, WRE achieved the best performance in the prediction of cell viability. Furthermore, it can be found that the predictive performance of all three feature selection methods gradually improved with the increase in drug concentration. Meanwhile, we verified the feature selection method WRE based on the CCLE-O'Neil dataset (Fig. 1C). Among the three feature selection models, WRE tended to make better predictions for most drugs. The key features selected by WRE were ranked according to their importance scores among different drug concentrations (Table S1). Drug properties accounted for most of the top ten key features at all drug concentrations. In addition, the number of features selected by our method increased with the increase in drug concentration and the prediction performance of the model became gradually stable. To a certain degree, it could explain the phenomenon that the prediction performance of the model became better with the increase in drug concentration. To gain a deeper understanding of the biological functions of the selected genes, we analyzed the selected characteristic genes when drug concentration equaled 2.1 μmol/L. Figure 1D and S4B present the enrichment analysis of selected 289 genes using the KEGG data set. The two enrichment pathways with the highest significance, mucin-type O-glycan biosynthesis and other types of O-glycan biosynthesis were both related to O-linked glycosylation, which is one of the important characteristics of tumors, involved in all aspects of tumor occurrence and metastasis.^1^

**Figure 1:**
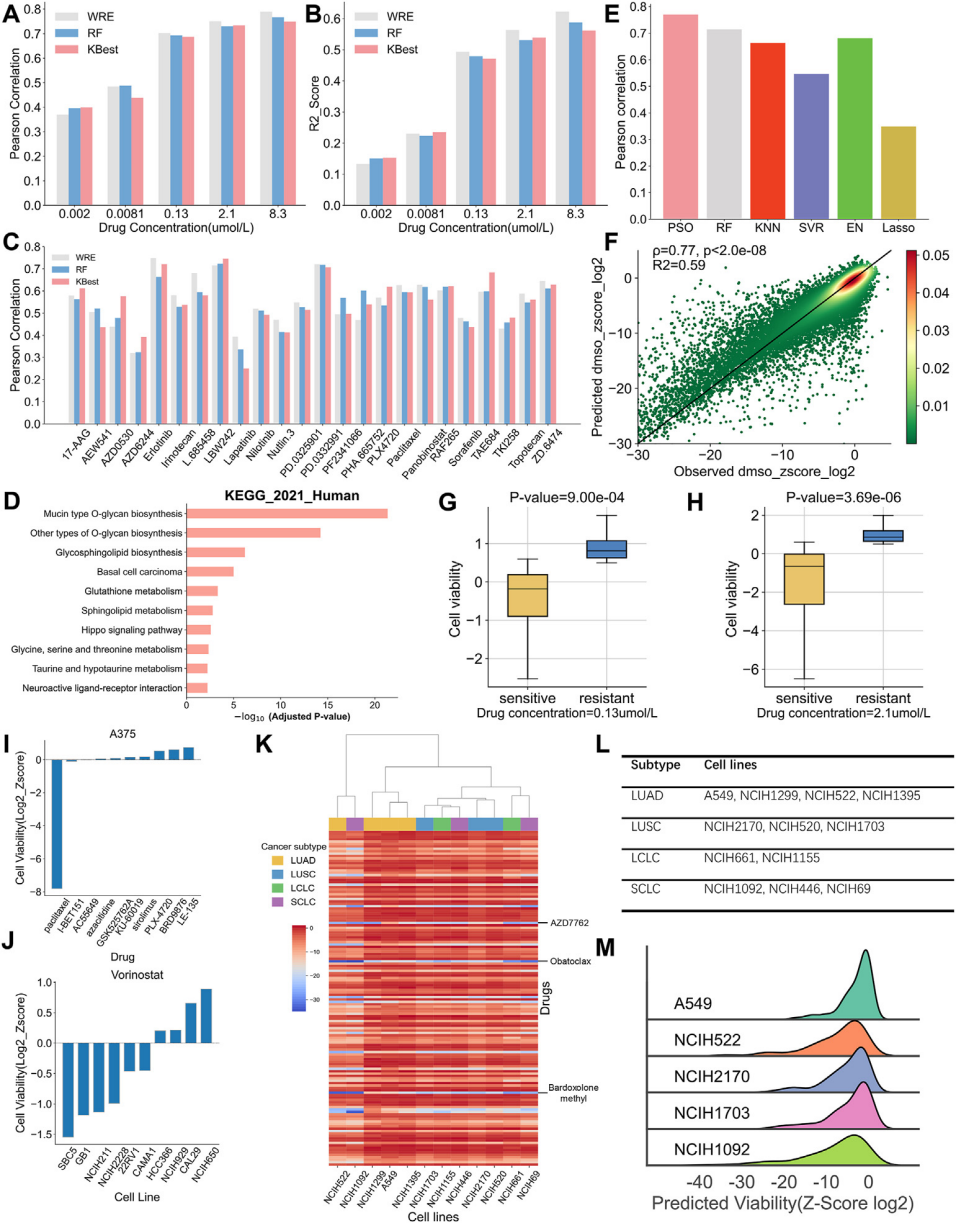
Performance comparison and analysis of the WRE-XGBoost model and its prediction on lung cancer cell lines. **(A)** The bar plot of Pearson's correlation coefficients using three feature selection methods in the COSMIC-CTRP dataset. **(B)** The bar plot of the coefficient of determination using three feature selection methods in the COSMIC-CTRP dataset. **(C)** The bar plot of Pearson's correlation coefficients using three feature selection methods in the CCLE-O'Neil dataset. **(D)** Enrichment analysis of screened genomic features in the COSMIC-CTRP dataset (drug concentration = 2.1 umol/L) **(E)** Comparison of drug response prediction across six machine learning algorithms in the COSMIC-CTRP dataset. **(F)** The scatter plot demonstrating the performance of WRE-XGBoost in the COSMIC-CTRP dataset. **(G, H)** The bar plot presenting the comparison of the sensitive drug group with the resistant drug group in the COSMIC-CTRP dataset. **(I)** The bar plot presenting the cell viability of the cell line A375 in different drugs. **(J)** The bar plot presenting the cell viability of the drug vorinostat in different drugs. **(K)** The heatmap presenting predicted cell viability (Z-score log2) for 140 drugs across 12 lung cancer cell lines. **(L)** The cell lines selected from four subtypes of lung cancer. **(M)** The ridge plot presenting the overall distribution of predicted cell viability (Z-score log2) across five lung cancer cell lines.

Further, we made comparisons with existing machine learning methods including RF, k-NearestNeighbor (KNN), support vector regression (SVR), elastic net (EN), and lasso regression (Lasso) to evaluate the performance of the WRE-XGBoost model. Figure 1E presents the distribution of Pearson's correlation coefficients under the condition of drug concentration = 8.3 μmol/L. WRE-XGBoost obtained the highest correlation, closely followed by RF. At the same time, we pooled our predictions across all drugs and cell lines and obtained a Pearson's correlation coefficient of 0.77 between predicted and observed cell viabilities (R^2^ = 0.59; *P* < 2.0e-08) (Fig. 1F). To determine whether sensitive and resistant drugs affect cell viability in a significant manner, we binarized the drug sensitivity data according to mean variance. We first normalized the data to zero mean, and the mean value of the data became zero. We then binarized the data based on variance, dividing the dataset into two groups on average, sensitive drugs and resistant drugs. A drug was defined as sensitive if it was below the mean variance of cell viability minus 0.5 (viability≤Var(viability)−0.5) and as resistant if it was above the mean variance minus 0.5 (viability≥Var(viability)−0.5). Figure 1G and H present the significance levels of prediction when the drug concentrations equaled 0.13 μmol/L and 2.1 μmol/L. The results showed that there were significant differences in the mean values of distinguishing sensitive drugs from resistant drugs using the predicted model. Similarly, we also verified the machine learning method using the CCLE-O'Neil dataset (Fig. S2). Our model achieved the best prediction results in 11 of 24 CCLE anti-cancer drugs. The results showed that our method can significantly improve the performance of drug response prediction. In addition to cell viability prediction through the predictive models, we checked the consistency of the predicted results with the literature (Fig. 1I, J). From Figure 1I, we found that the cell viability of the A375 cell line belonging to melanoma under the action of paclitaxel was much lower than other drugs. Studies have confirmed that the growth of melanoma cells can be effectively inhibited by paclitaxel combined with Icariside II at a clinically acceptable concentration and paclitaxel has great potential for the treatment of melanoma.^2^ The high sensitivity of NCIH211 and NCIH228, both small cell lung cancer cell lines, to vorinostat indicated inhibition. Researchers found that vorinostat combined with cisplatin can reduce the toxicity of small-cell lung cancer and reduce the adverse reactions of the drug (Fig. 1J).^3^

Finally, the lung cancer cell lines were used to verify our possibility of inferring drugs in precision oncology based on WRE-XGBoost prediction. Lung cancer clinically includes non-small cell lung cancer and small cell lung cancer. Non-small cell lung cancer is further divided into lung adenocarcinoma, lung squamous cell carcinoma, and large cell lung carcinoma. Small cell lung cancer can only be clinically classified to a limited extent and is the most malignant cancer in lung cancer, so it was not classified further in our experiment. We predicted drug response for each of 12 cell lines from four subtypes of lung cancer for 140 drugs (Fig. 1L and Table S2) in the COSMIC-CTRP dataset (Fig. 1K). Different subtypes of lung cancer cell lines achieved certain accuracy in clustering in which lung adenocarcinoma and lung squamous cell carcinoma could be basically clustered together. According to Figure 1K, M, NCIH522 and NCIH1092 were predicted to be the most sensitive to these drugs and A549 was predicted to be the least sensitive. From the heatmap distribution, we found three drugs that were more effective for the drug response of comprehensive lung cancer cell lines: AZD7762, obatoclax, and bardoxolone methyl. CHK1 inhibitor AZD7762 was significantly correlated with lepidic score, and the regulation of DNA damage and cell cycle response by the CHK1 pathway was considered to be one of the reasons for the therapeutic resistance of lung cancer.^4^ Obatoclax induced apoptosis in multiple small cell lung cancer cell lines and it was considered to be a drug regimen used to activate cell lines before chemotherapy.^5^ The experimental results of our model can be correlated with the literature on anti-cancer drugs to prove the predictive effects.

In summary, this study proposes the algorithm WRE-XGBoost to predict cell line viability based on gene expression and drug properties. The use of these predictions to prioritize promising drugs for clinical application can reduce the time and cost of discovering newly available drugs.

## Author contributions

Y.J. conducted the experiments, performed the data analysis, and wrote the manuscript. Y.Q. designed the study and supervised the research. M.C. contributed a critical review and polished the language of the manuscript. Z.X. provided some of the materials and experimental suggestions. All authors read and approved the final manuscript.

## Conflict of interests

The authors declared that they had no competing interests.

## Funding

This work was supported in part by the Shanghai Science and Technology Innovation Action Planning (No. 20dz1203800 to M.C.), the Research and Development Planning in Key Areas of Guangdong Province, China (No. 2021B0202070001 to M.C.), and the National Natural Science Foundation of China (No. 61702325 to Y.Q.).

## Figures and Tables

**figs1:**
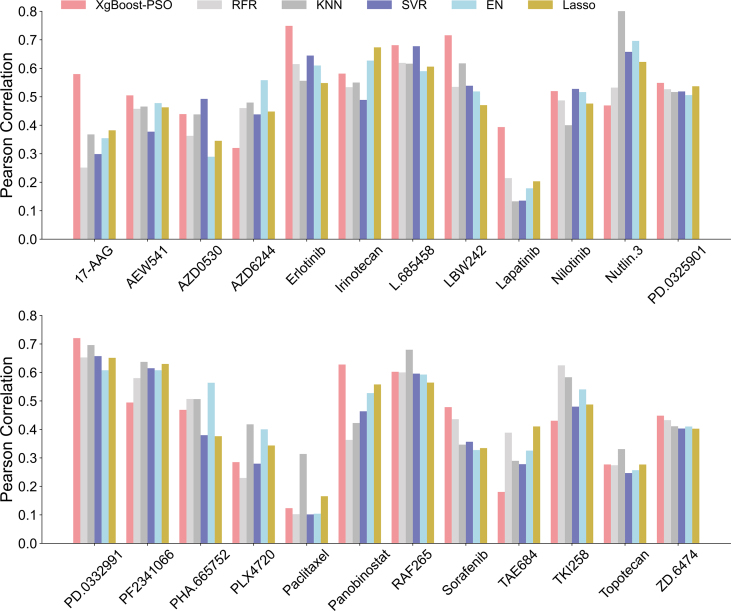


**figs2:**
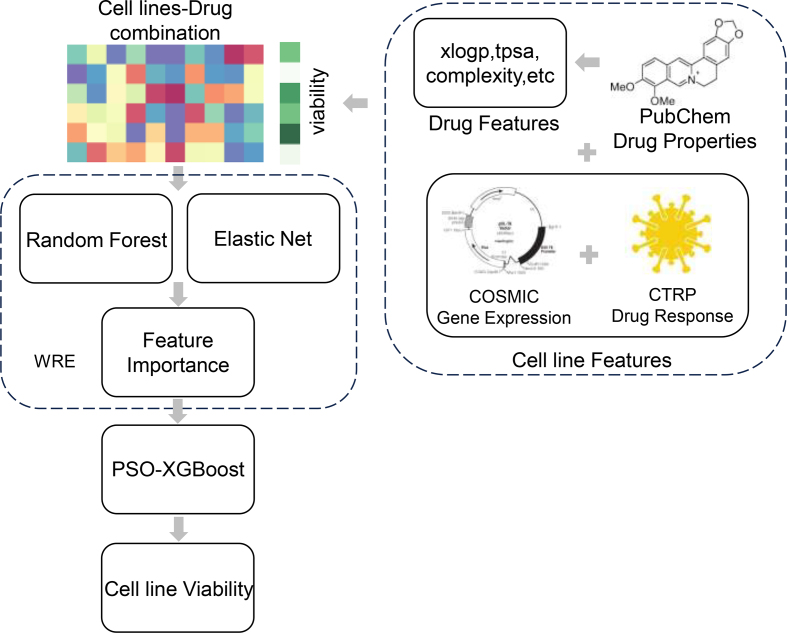


**figs3:**
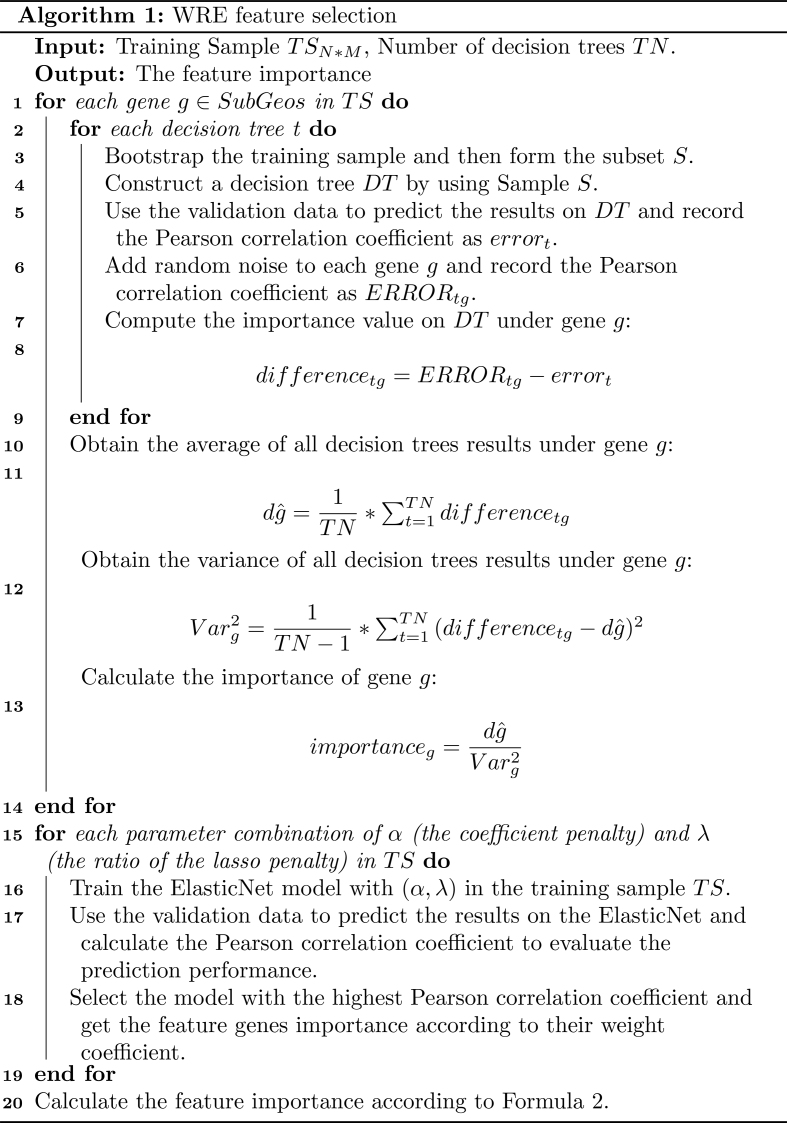


**figs4:**
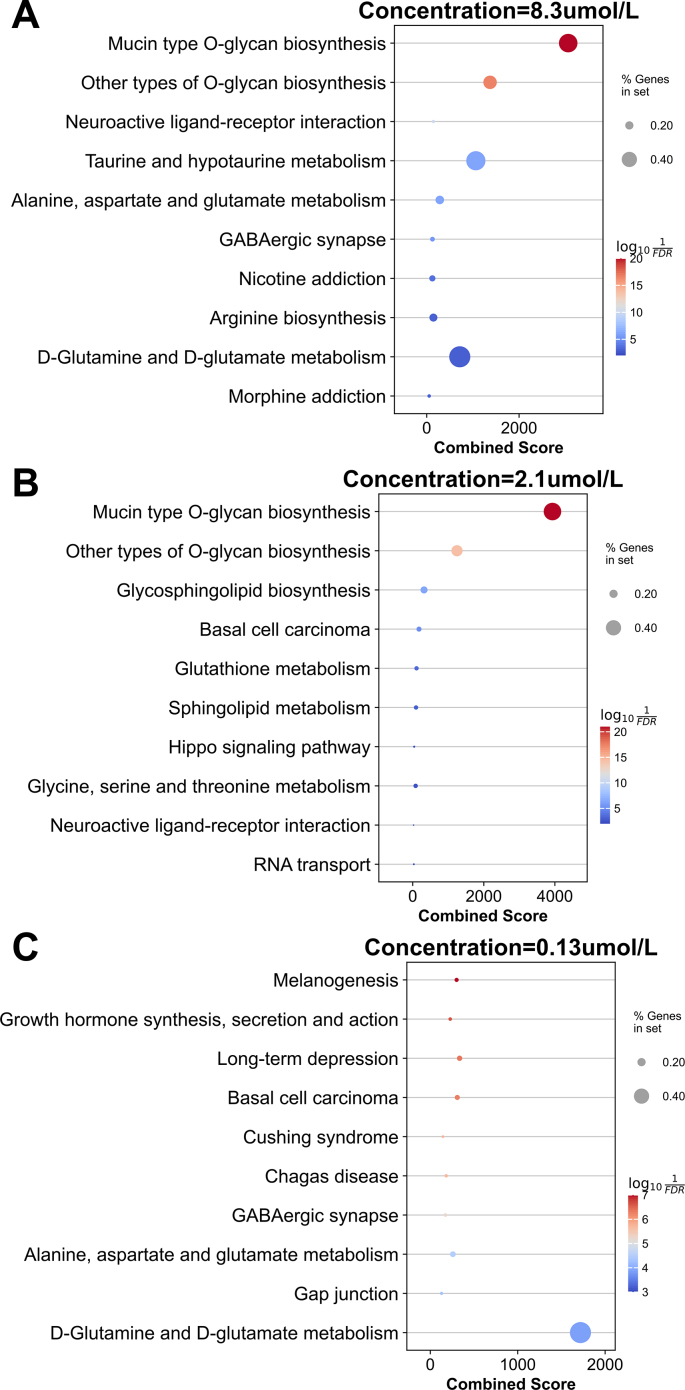


**figs5:**
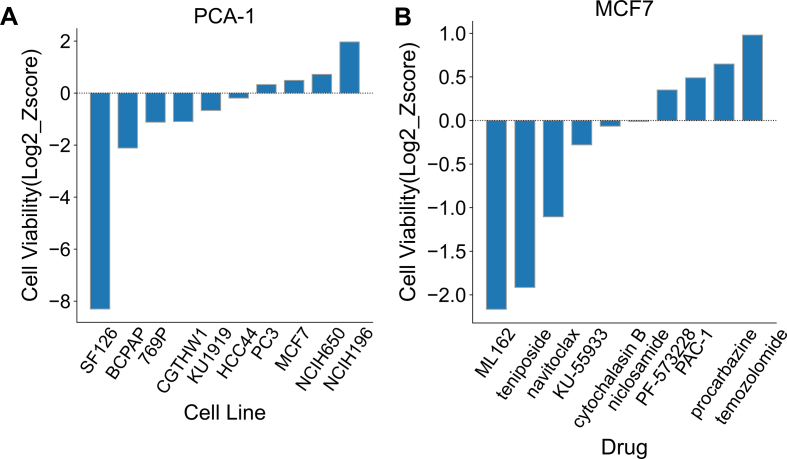


## References

[1] Pinho S.S., Reis C.A. (2015). Glycosylation in cancer: mechanisms and clinical implications. Nat Rev Cancer.

[2] Wu J., Guan M., Wong P., Yu H., Dong J., Xu J. (2012). Icariside II potentiates paclitaxel-induced apoptosis in human melanoma A375 cells by inhibiting TLR4 signaling pathway. Food Chem Toxicol..

[3] Pan C.H., Chang Y.F., Lee M.S. (2016). Vorinostat enhances the cisplatin-mediated anticancer effects in small cell lung cancer cells. BMC Cancer.

[4] Nguyen T.T., Lee H.S., Burt B.M. (2022). A lepidic gene signature predicts patient prognosis and sensitivity to immunotherapy in lung adenocarcinoma. Genome Med.

[5] Dean E.J., Cummings J., Roulston A. (2011). Optimization of circulating biomarkers of obatoclax-induced cell death in patients with small cell lung cancer. Neoplasia.

