# Supplementary material for: Predicting anti-cancer drug sensitivity through WRE-XGBoost algorithm with weighted feature selection
Source: Genes Dis. 2024 Mar 22;12(2):101275. doi: 10.1016/j.gendis.2024.101275 (PMC11585671; doi:10.1016/j.gendis.2024.101275)
Supplement: Multimedia component 1 [file mmc1.docx]

**Predicting anticancer drug sensitivity through WRE-XGBoost algorithm with weighted feature selection**

Yiyao Jiang^a,b^, Ming Chen^a,b^, Zhongmin Xiong^a,b,**^ and Yufang Qin^a,b,*^

^a^ College of Information Technology, Shanghai Ocean University, Shanghai, 201306, China

^b^ Key Laboratory of Fisheries Information Ministry of Agriculture, Shanghai, 201306, China

^**^Corresponding author.

^*^Corresponding author.

E-mail addresses: yfqin@shou.edu.cn (Y. Qin), zmxiong@shou.edu.cn (Z. Xiong)

**Additional Background**

Anti-tumor targeted therapy based on genomic features can greatly improve the survival rate of cancer patients and has less side effects than traditional therapy. With the accumulation of drug screening and multi-omics data, many attempts based on machine learning techniques have been made to predict drug sensitivity. Riddick et al. developed an ensemble regression model using random forest to predict drug sensitivity based on gene expression data of NCI-60 cell lines.^1^ The model was also used to create drug-specific gene expression signatures and identify core cell lines associated with each drugs’ response. Rahman et al. integrated tumor heterogeneity into random forest and proposed Heterogeneity Aware Random Forests (HARF).^2^ When the average drug response of cancer types was different, the prediction results were better than the traditional random forest. Also based on gene expression data, Dong et al. used support vector machine (SVM) to predict drug sensitivity accurately using baseline gene expression of cell line panels from preclinical studies.^3^ Wang et al. added the pathway activity score to the elastic net, and emphasized the ability of the pathway-based model to reveal the drug mechanism.^4^ Bayesian multi-view multi-task linear regression model used the genomic information encoded in Functional-Linked-Networks of genes (FLNs) form to enhance the understanding of drug mechanism and identified new combinations of biomarkers.^5^

At present most computational models for predicting drug sensitivity of cancer cell lines involve gene-level characteristics, helping reveal new drug mechanisms. But researchers found that the reproducibility of gene expression in the study was limited.^6^ Gerdes et al. developed Drug Ranking Using ML (DRUML) model and proved that the large-scale proteomics and phosphoproteomics data can be used as the input of the model to predict the drug response with low error.^7^ The verification results of independent data sets indicated that DRUML ranked anticancer drugs accurately according to their efficacy in various pathologies. In the NCI/Dream7 Challenge, the BEMKL method found that gene expression microarray always provides the best data for individual analysis and improved the signal-to-noise ratio and the prediction performance of the model by capturing common signal between multiple omics profiles (Gene expression, RNA-seq, Exome, Methylation, RPPA and CNV).^8^ Similarly, the cwKBMF methods extended the state-of-the-art kernelized Bayesian matrix factorization (KBMF), improved the prediction performance by combining the path with prior knowledge and inferred the relationship between pathway and drug response.^9^ Based on the chemical structural similarity between drugs, Zhang et al. proposed a two-layer network model which effectively captured the interaction between cell lines and drugs, and was not affected by the huge dimensions of gene expression and copy number variation.^10^ In addition, the auxiliary information of drugs has been proved to improve the prediction accuracy by providing a priori information for drug response.^11^ Besides the gene expression of cell lines, we also considered the auxiliary information of drugs as the input characteristics for the predictive model.

In this work, we developed WRE-XGBoost model, using gene expression and drug properties as input, which consists of a weighted algorithm of the random forest regression and elastic net regression (WRE) to select the important features and an improved XGBoost algorithm associating with PSO to predict the cell line viability.

**Materials and Methods**

**Data preprocessing**

We used five datasets in this work, including the Catalogue of Somatic Mutations in Cancer (COSMIC), the Cancer Therapeutics Response Portal (CTRP), the Cancer Cell Line Encyclopedia (CCLE), the drug response dataset from O’Neil et al. and PubChem. COSMIC contains 16,248 gene expression data of 971 cell lines treated with Z-score and can be downloaded from <https://cancer.sanger.ac.uk/cell_lines/download>.^12^ CTRPv2 covered drug reactions of 481 compounds on 860 cell lines and we selected log2-transformed and z-scored cell viability as a target for our modeling, which could be downloaded from the following weblink: <http://portals.broadinstitute.org/ctrp/>.^13^ PubChem(<https://pubchem.ncbi.nlm.nih.gov>) is a popular repository of chemical structures and their activities.^14^ The PubChem Bioassay database currently contains results for the bioactivity of over 700,000 compounds. We selected 17 chemical properties of the compounds as the auxiliary information of drug (**Table S3**). The above three datasets were merged to a dataset, containing 288 drugs, 116 cell lines and the cell viability of drugs acting on cell lines, which was denoted by COSMIC-CTRP dataset (**Table S4**). For the sake of studying the relationship between the accuracy of the cell viability prediction model and drug concentration, we divided the COSMIC-CTRP dataset into five subsets with same size according to the drug concentrations: 0.002umol/L, 0.0081umol/L, 0.13umol/L, 2.1umol/L and 8.3umol/L.

To verify the effectiveness of our prediction model, we also accessed the Cancer Cell Line Encyclopedia (CCLE) and the O’Neil dataset as validation datasets. Raw gene expression was collected from CCLE (<https://sites.broadinstitute.org/ccle>) for 24 drugs.^15^ The O’Neil dataset consists of the response of 39 cancer cell lines and 38 compounds, which could be downloaded from website <https://mct.aacrjournals.org/content/15/6/1155.figures-only>.^16^ We selected 39 cell lines and 24 compounds contained in two datasets, and then integrated them into CCLE- O’Neil dataset (**Table S5**).

**Model establishment**

The workflow of the proposed WRE-XGBoost model is shown in **Fig. S2**. First, the gene expression level of cell line and the properties of drug are as the input data. Then, the weighted algorithm of the random forest regression and elastic net regression (WRE) is used to select key features. Finally, the selected features are input into XGBoost associating with PSO for predicting drug sensitivity.

**Feature selection**

For each cell line-drug pair in COSMIC-CTRPP dataset, it has 16,248 gene expression features and 17 drug property features. Random forest and Elastic Net were used in WRE-XGBoost model. On each leaf node of the decision tree, we randomly selected features from the expression gene space $SubGeos=\left( g_{1},g_{2},g_{3},{,g}_{m} \right)$ and drug property space $SubDrugs=\left( d_{1},d_{2},d_{3},{,d}_{n} \right)$ to form a subspace, obtained the optimal gene segmentation results through weak decision trees and averaged predictions of the classifier to get final result. Random numbers in $\left[ -1,1 \right]$ were added to the test set as noise to increase the robustness of the training model to noise. The difference and degree matrix of the prediction results by the random forest regression before and after the test set was added with the noise was calculated. We ranked all the features according to the ratio of the degree matrix to the standard deviation and recorded the importance order of features and the features screened out. The pseudo code flow of the algorithm is shown in **Fig. S3.**

Then we use elastic net regression to select the key features. The penalty function is represented by

$J\left( \theta\right)=MSE\left( \theta\right)+\alpha\lambda\sum_{i=1}^{n} \left| \theta_{i} \right|+\frac{1-\alpha}{2}\lambda\sum_{i=1}^{n} \theta_{i}^{2}$ (1)

where $\alpha$ and $\lambda$ determine the contribution value of ridge regression and lasso regression. We selected the mixed parameter $\alpha$ and $\lambda$ of the elastic net regression and obtained the importance order of features by Pearson correlation coefficient of the validation set.

We presented a weighted algorithm of the random forest regression and elastic net regression (referred as WRE) to select key features. ${WRE}_{Rank}$ is used to represent the importance score of each feature. The higher the rank of the importance of a feature, the greater its role in drug response prediction.

${WRE}_{Rank}=\frac{e^{RFPearson}*{RF}_{Rank}+e^{ENPearson}*{EN}_{Rank}}{e^{RFPearson}+e^{ENPearson}}$ (2)

where $RFPearson$ and $ENPearson$ are the Pearson correlation of the validation set using the random forest regression and elastic net regression. ${RF}_{Rank}$ and ${EN}_{Rank}$ are the importance order of features selected by the two algorithms respectively.

**Cell viability prediction algorithm**

In our work, we used eXtreme Gradient Boosting (XGBoost) to predict cell viability and obtained prediction scores for each leaf node in a decision tree. Through multiple iterations, the weak estimates of all rounds were summed to obtain the predicted results ${cd}_{i}$for the $i$-th cell line-drug pair as follows:

${cd}_{i}=\sum_{k=1}^{K} f_{k}({sample}_{i})$ (3)

where $f_{k}({sample}_{i})$ is the prediction score on the $k$-th decision tree for the $i$-th cell line-drug pair on the selected feature, *K* is the number of decision trees.

Particle Swarm Optimization (PSO) can find the global optimal solution of the problem through the cooperation among groups. In order to choose the optimal parameters of XGBoost, we use particle swarm optimization to solve the complex global optimization problem. We first initialized the parameters of particle swarm, encoded parameters as binary numbers and then transformed the prediction into a discrete combinatorial optimization problem. For each iteration, the Pearson’s correlation coefficient and fitness of each particle were calculated in parallel and compared to the global best position. If the current fitness value was higher, the global best position would be replaced.

At the same time, in order to overcome the shortcoming of premature convergence of particle swarm, we introduced dynamic weight adjustment.

$\omega\left( k \right)=\alpha_{1}e^{\frac{-\varphi*k}{T}}+\alpha_{2}e^{\frac{\varphi*k}{T}}$ (4)

where *T* is the maximum number of iterations, *k* presents the current number of iterations, $\alpha_{1}=\frac{\omega_{2}e^{\varphi}-\omega_{1}e^{2\varphi}}{1-e^{2\varphi}}{, \alpha_{2}=\frac{\omega_{1}-\omega_{2}e^{\varphi}}{1-e^{2\varphi}}}$, $\omega_{1}$ and $\omega_{2}$ are the minimum inertia weight and maximum inertia weight.

**Additional Results**

**Feature ranking**

According to the key features selected by WRE in **Table S1**, Xlogp and defined atom stereo were the first two most important features for four drug concentrations. Xlogp is a measure of the hydrophobicity of a substance, indicating the magnitude of its lipophilicity. Weak water solubility is one of the main challenges faced by clinical application of chemotherapeutic drugs.^17^ Many anticancer drugs need to be combined with harmful solvents because of their hydrophobicity and poor pharmacokinetics.^18^ An atom or a group of atoms is considered to be a stereocenter, also known as a chiral center, if the interchange of two connected ligands yields a new stereoisomer. Chiral drug is a concern at all stages from drug design to clinical application due to the corresponding selectivity of protein at binding.^19^ Therefore we could verify the validity of the selected features.

**Enrichment analysis**

Kegg (Kyoto encyclopedia of genes and genomes) database is a database that systematically analyzes gene functions and links genomic information and functional information, including pathway database, gene database and genome database.^20^ **Fig. S4** presents the bubble plot generated by using the KEGG dataset to perform enrichment analysis on the three subsets with different concentrations. Under the action of high concentration drugs (concentration=8.3mol/L and 2.1mol/L), enriched genes are concentrated in O-glycans and the enrichment significance is high (**Fig. S4 A, B**). The non-essential amino acid glutamate is an excitatory neurotransmitter involved in carcinogenic signaling pathways, and also a bioenergy substrate for the proliferation for normal and cancer cells.^21^ When the drug concentration was 0.13mol/L, the selected genes were largely enriched in D-glutamine and D-glutamate metabolism (**Fig. S4C**).

**Prediction results of drugs action on cell lines**

From **Fig. S5A**, we found that under the action of PCA-1, the human brain tumor cell SF126 exhibited the lowest cell survival rate and was far lower than other cell lines. At the same time, multiple drugs such as ML162 and teniposide exerted similar inhibitory effects on the MCF7 cell line (**Fig. S5B**). Studies have shown that teniposide loaded micelles have improved hydrophilic and therapeutic efficiency and can effectively inhibit the growth of breast cancer cell line MCF7 in vivo.^22^

**References**

1. Riddick G, Song H, Ahn S, et al. Predicting in vitro drug sensitivity using Random Forests. *Bioinformatics.* 2010;27(2):220-224.

2. Rahman R, Matlock K, Ghosh S, Pal R. Heterogeneity Aware Random Forest for Drug Sensitivity Prediction. *Scientific Reports.* 2017;7(1):11347.

3. Dong Z, Zhang N, Li C, et al. Anticancer drug sensitivity prediction in cell lines from baseline gene expression through recursive feature selection. *BMC Cancer.* 2015;15:489.

4. Wang X, Sun Z, Zimmermann MT, Bugrim A, Kocher J-P. Predict drug sensitivity of cancer cells with pathway activity inference. *BMC Medical Genomics.* 2019;12(1):15.

5. Ammad-ud-din M, Khan S, Wennerberg K, Aittokallio T. Systematic identification of feature combinations for predicting drug response with Bayesian multi-view multi-task linear regression. *Bioinformatics (Oxford, England).* 2017;33:i359-i368.

6. Ein-Dor L, Zuk O, Domany E. Thousands of samples are needed to generate a robust gene list for predicting outcome in cancer. *Proceedings of the National Academy of Sciences.* 2006;103(15):5923-5928.

7. Gerdes H, Casado P, Dokal A, et al. Drug ranking using machine learning systematically predicts the efficacy of anti-cancer drugs. *Nature Communications.* 2021;12.

8. Costello JC, Heiser LM, Georgii E, et al. A community effort to assess and improve drug sensitivity prediction algorithms. *Nature Biotechnology.* 2014;32(12):1202-1212.

9. Ammad-ud-din M, Khan SA, Malani D, et al. Drug response prediction by inferring pathway-response associations with kernelized Bayesian matrix factorization. *Bioinformatics.* 2016;32(17):i455-i463.

10. Zhang N, Wang H, Fang Y, Wang J, Zheng X, Liu XS. Predicting Anticancer Drug Responses Using a Dual-Layer Integrated Cell Line-Drug Network Model. *PLoS Comput Biol.* 2015;11(9):e1004498.

11. Yang M, Simm J, Lam CC, et al. Linking drug target and pathway activation for effective therapy using multi-task learning. *Scientific Reports.* 2018;8(1):8322.

12. Forbes SA, Bindal N, Bamford S, et al. COSMIC: mining complete cancer genomes in the Catalogue of Somatic Mutations in Cancer. *Nucleic Acids Research.* 2010;39(suppl_1):D945-D950.

13. Rees MG, Seashore-Ludlow B, Cheah JH, et al. Correlating chemical sensitivity and basal gene expression reveals mechanism of action. *Nat Chem Biol.* 2016;12(2):109-116.

14. Wang Y, Xiao J, Suzek TO, Zhang J, Wang J, Bryant SH. PubChem: a public information system for analyzing bioactivities of small molecules. *Nucleic Acids Research.* 2009;37(suppl_2):W623-W633.

15. Ghandi M, Huang FW, Jané-Valbuena J, et al. Next-generation characterization of the Cancer Cell Line Encyclopedia. *Nature.* 2019;569(7757):503-508.

16. O'Neil J, Benita Y, Feldman I, et al. An Unbiased Oncology Compound Screen to Identify Novel Combination Strategies. *Molecular cancer therapeutics.* 2016;15(6):1155-1162.

17. Karaosmanoglu S, Zhou M, Shi B, Zhang X, Williams G, Chen X. Carrier-free nanodrugs for safe and effective cancer treatment. *Journal of Controlled Release.* 2020;329.

18. Xu MQ, Zhong T, Yao X, et al. Effect of XlogP and hansen solubility parameters on the prediction of small molecule modified docetaxel, doxorubicin and irinotecan conjugates forming stable nanoparticles. *Drug Delivery.* 2021;28:1603-1615.

19. Brooks WH, Guida WC, Daniel KG. The significance of chirality in drug design and development. *Current topics in medicinal chemistry.* 2011;11(7):760-770.

20. Kanehisa M, Araki M, Goto S, et al. KEGG for linking genomes to life and the environment. *Nucleic Acids Research.* 2007;36(suppl_1):D480-D484.

21. Stepulak A, Rola R, Polberg K, Ikonomidou C. Glutamate and its receptors in cancer. *Journal of Neural Transmission.* 2014;121(8):933-944.

22. Chu B, Shi S, Li X, et al. Preparation and evaluation of teniposide-loaded polymeric micelles for breast cancer therapy. *International Journal of Pharmaceutics.* 2016;513(1):118-129.

**Supplementary Figures**

**
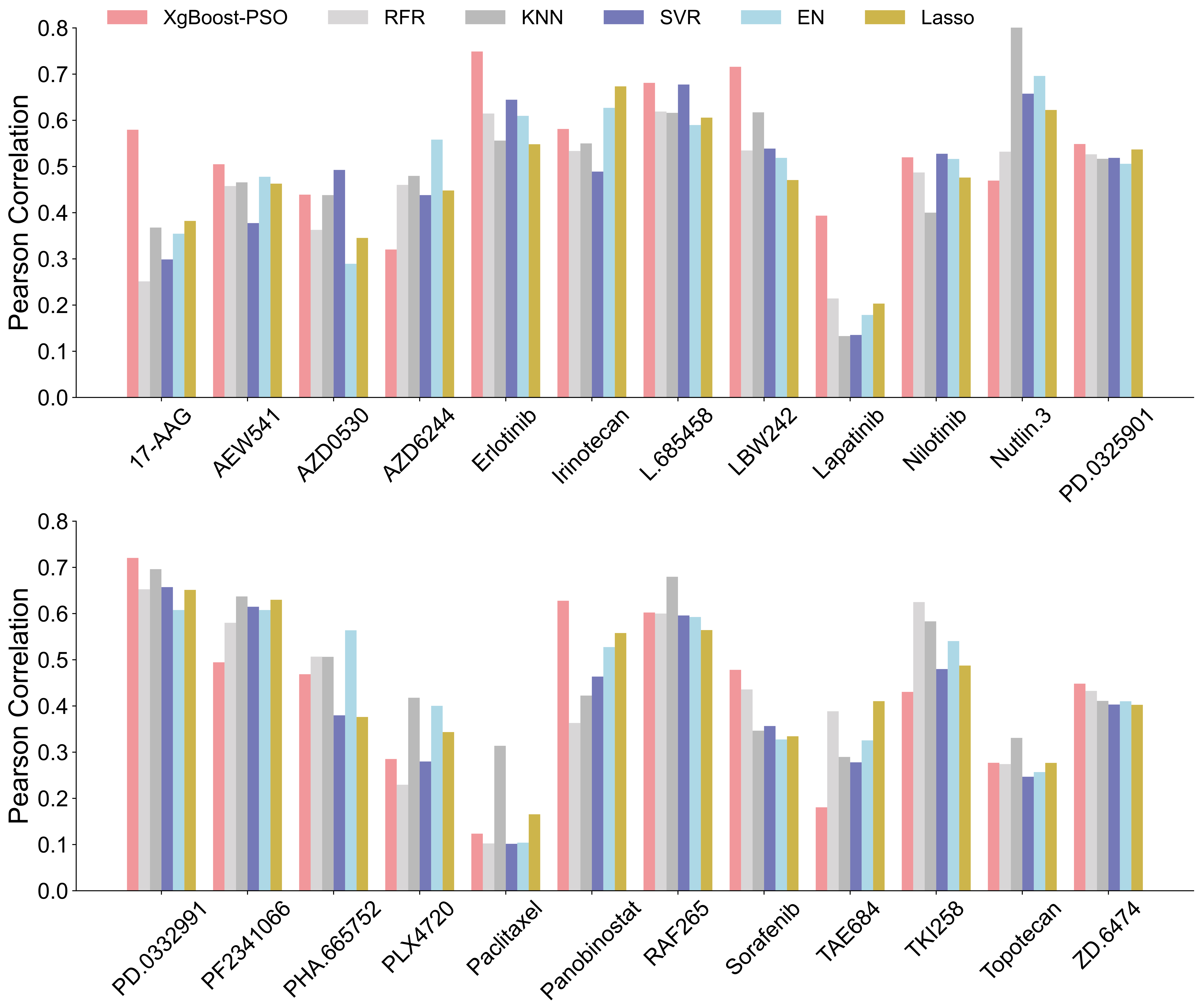
**

**Fig. S1** Barplot of Pearson’s correlation coefficients using six machine learning algorithms in the CCLE-O’Neil dataset.


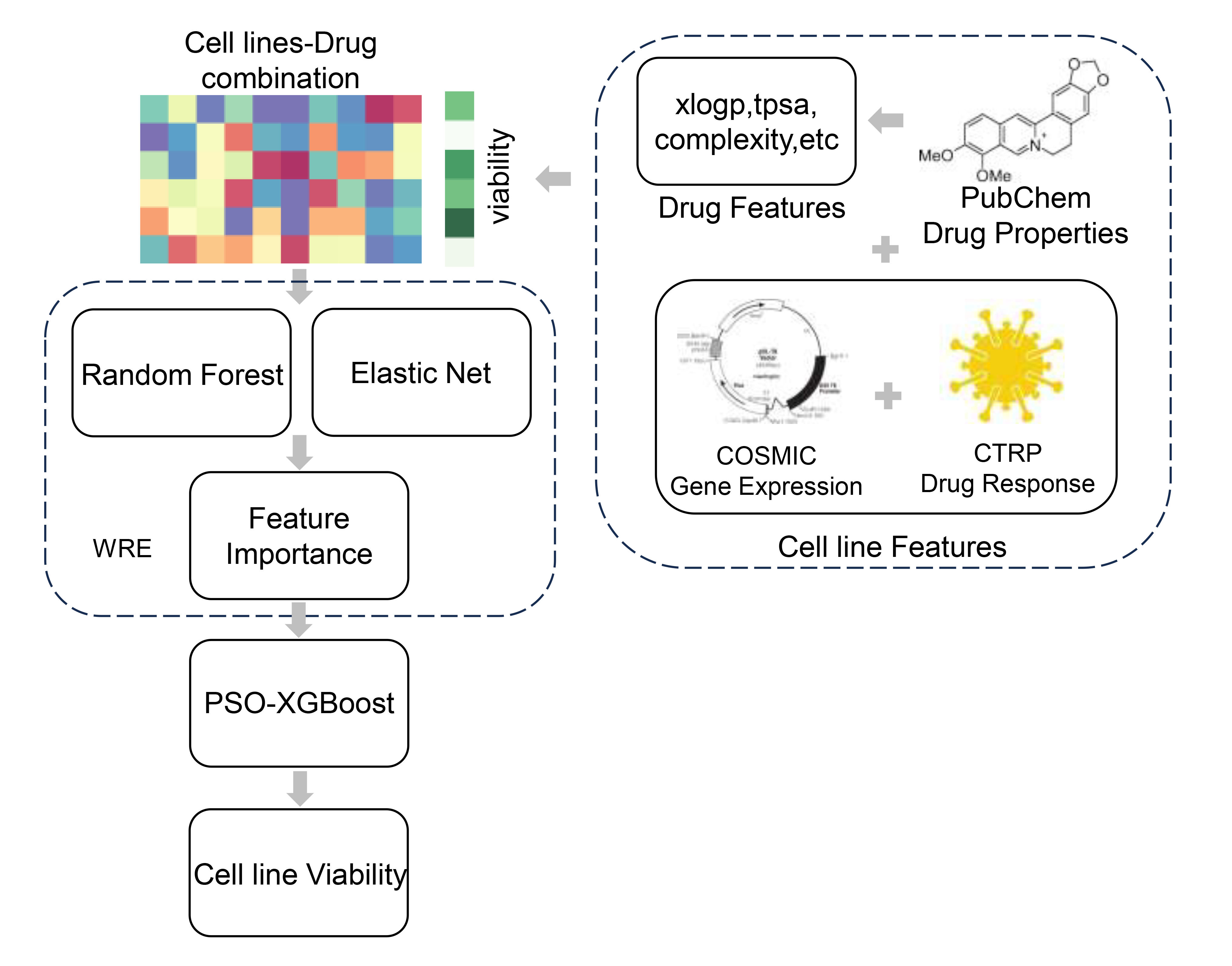


**Figure S2.** Workflow of WRE-XGBoost model.

**
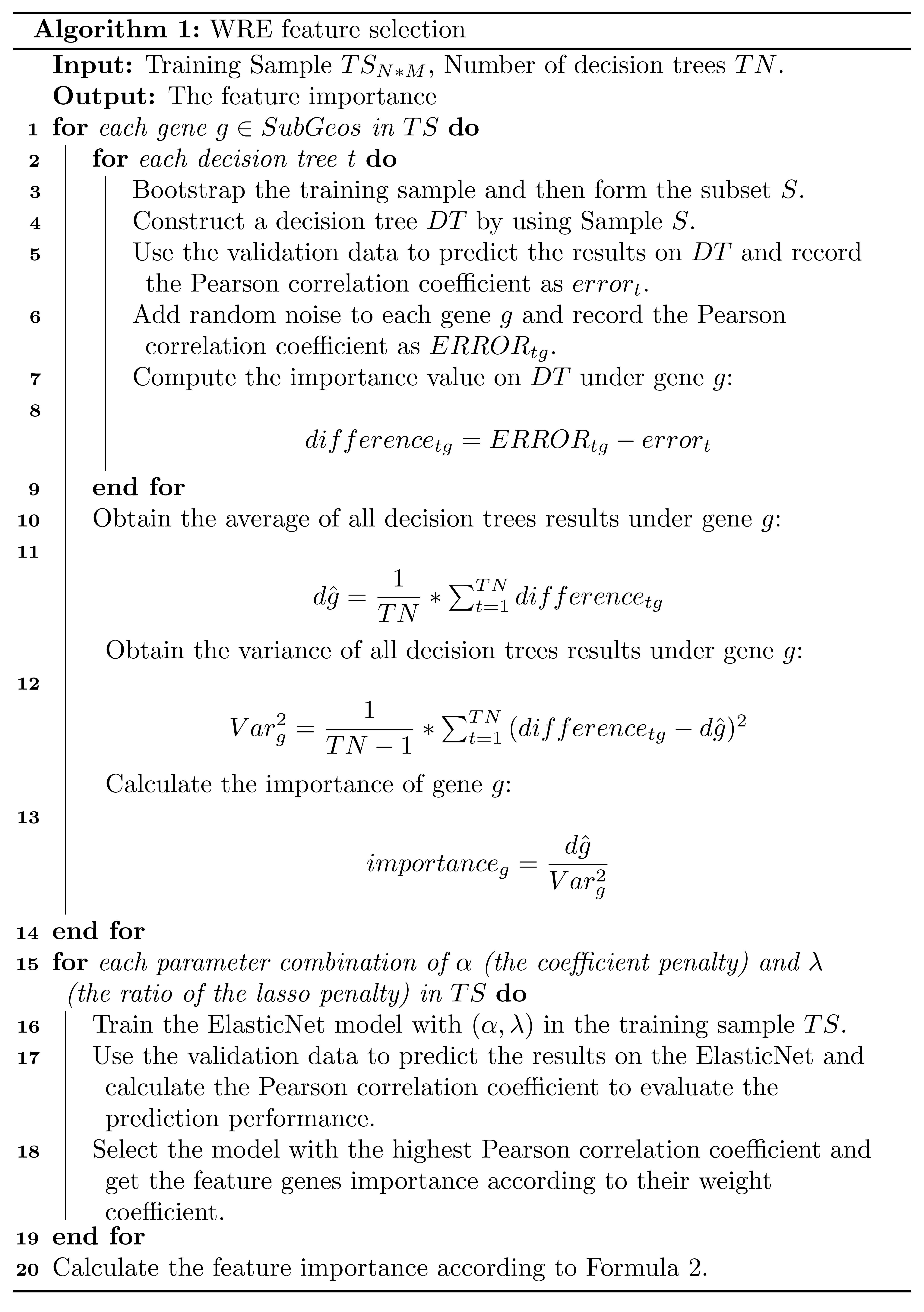
**

**Figure S3.** Pseudo code of WRE algorithm.

**
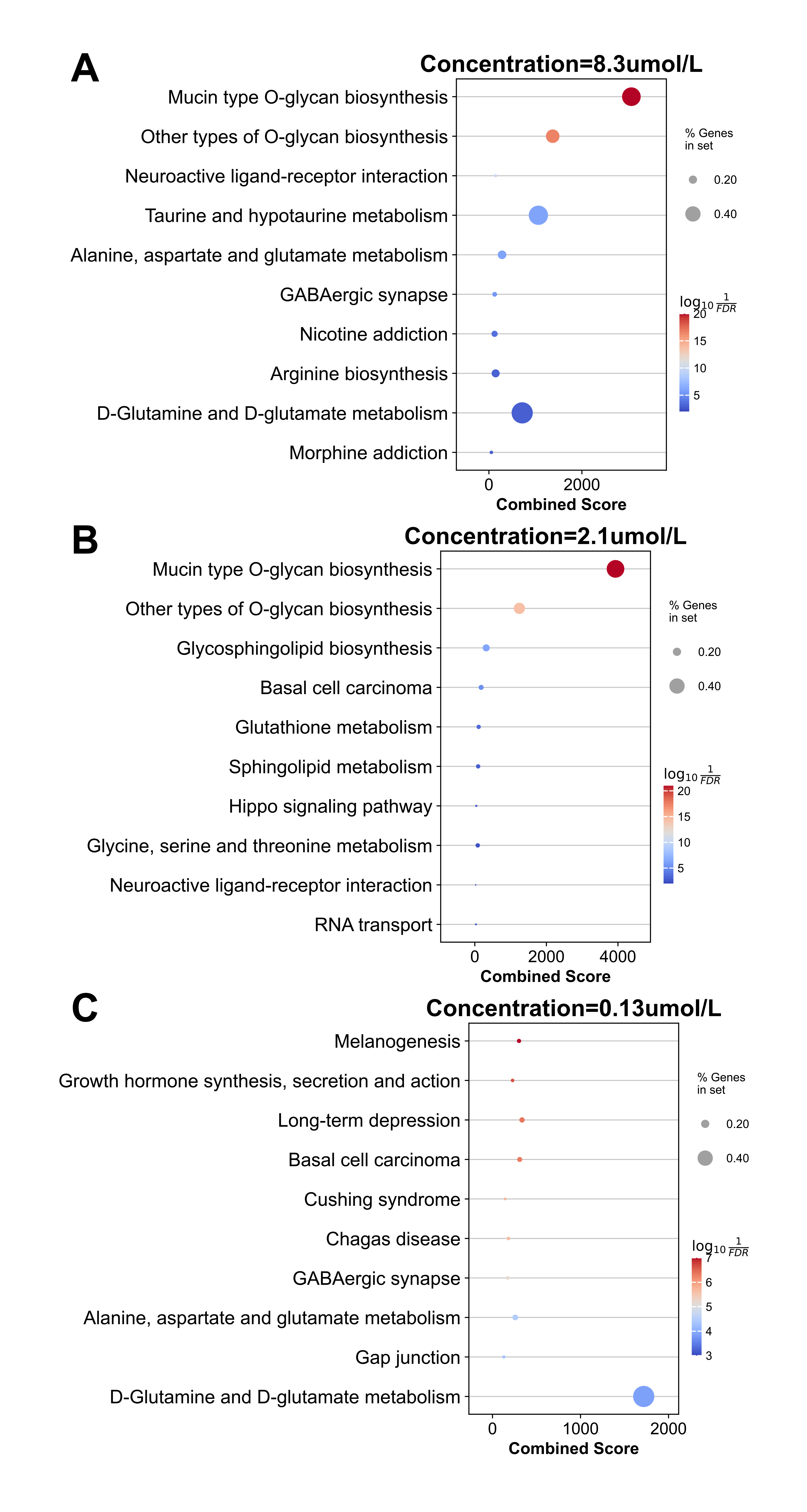
**

**Figure S4.** KEGG enrichment analysis of screened genomic features in COSMIC-CTRP dataset. The size of the bubble indicates the number of genes it contains. **A** Drug concentration=8.3mol/L. **B** Drug concentration=2.1mol/L. **C** Drug concentration=0.13mol/L.

**
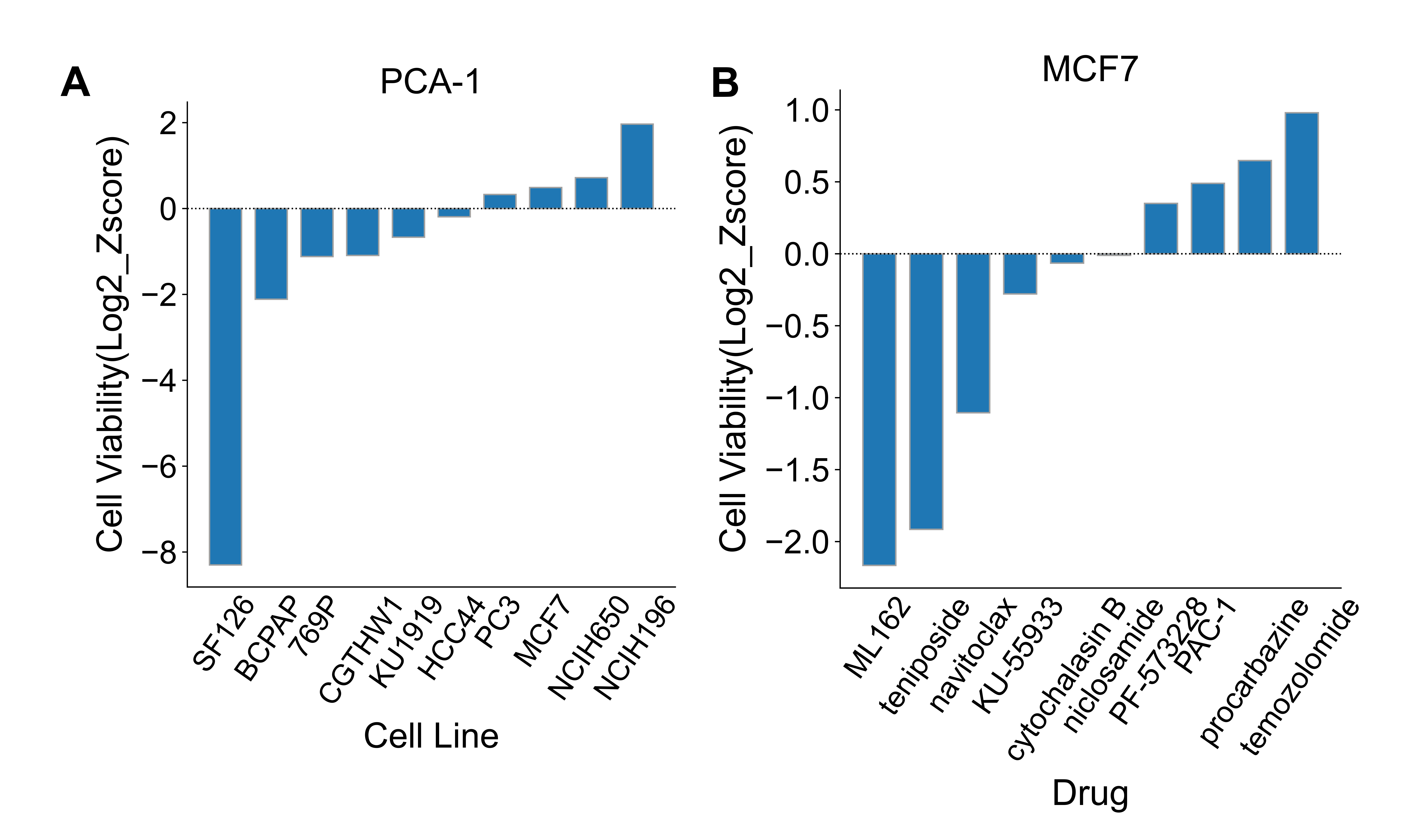
**

**Figure S5.** The predicted cell viability for different drugs and cell lines. **A** presented the cell viability of the drug PCA-1 in different cell lines. **B** presented the cell viability of the cell line MCF7 in different drugs.
